# Cryptococcal chest wall mass and rib osteomyelitis associated with the use of fingolimod: A case report and literature review

**DOI:** 10.3389/fmed.2022.942751

**Published:** 2022-09-07

**Authors:** Kent Carpenter, Ali Etemady-Deylamy, Victoria Costello, Mohammad Khasawneh, Robin Chamberland, Katherine Tian, Maureen Donlin, Brenda Moreira-Walsh, Emily Reisenbichler, Getahun Abate

**Affiliations:** ^1^Department of Internal Medicine, Saint Louis University, Saint Louis, MO, United States; ^2^SSM Saint Louis Network Microbiology, Saint Louis, MO, United States; ^3^Department of Pathology, Saint Louis University, Saint Louis, MO, United States; ^4^Department of Molecular Microbiology and Immunology, Saint Louis University, Saint Louis, MO, United States

**Keywords:** cryptococcus, fingolimod, chest mass, osteomyelitis, immunosuppression

## Abstract

Being introduced in 2010, fingolimod was among the first oral therapies for relapsing multiple sclerosis (MS). Since that time, postmarketing surveillance has noted several case reports of various cryptococcal infections associated with fingolimod use. To date, approximately 15 such case reports have been published. We present the first and unique case of cryptococcal chest wall mass and rib osteomyelitis associated with fingolimod use. The patient presented with left-side chest pain and was found to have a lower left chest wall mass. Computerized tomography (CT) showed chest wall mass with the destruction of left 7th rib. Aspirate from the mass grew *Cryptococcus neoformans*. The isolate was serotype A. Fingolimod was stopped. The patient received liposomal amphotericin B for 2 weeks and started on fluconazole with a plan to continue for 6–12 months. The follow-up CT in 6 weeks showed a marked decrease in the size of the chest wall mass. In conclusion, our case highlights the atypical and aggressive form of cryptococcal infection possibly related to immunosuppression from fingolimod use.

## Background

*Cryptococcus neoformans* is a common, encapsulated yeast from the *Tremellomycetes* class, known to commonly cause infections in immunosuppressed patients and rarely in immunocompetent patients (1). In immunocompetent patients, cryptococcal infection is often confined to the lungs that is commonly the primary infection site (2). This is mainly because of a robust Th1 immune response that is capable of controlling *C. neoformans* infection (2). In contrast, in immunocompromised patients, *C. neoformans* can cause severe disease with high mortality (3, 4). *C. neoformans* has capsular polysaccharides located externally to the cell wall with the flexibility to alter the composition, thereby altering antigenic properties and evading the immune response (5). The capsule is a virulence factor with immunomodulatory effects, affecting macrophages, neutrophils, and T cells. The capsule helps *C. neoformans* avoid phagocytosis, survive better in macrophages, and also induce widespread immunosuppression (6–10).

The most common presentations of cryptococcal infections include central nervous system (CNS) manifestations (meningoencephalitis), followed by pulmonary, and cutaneous manifestations (11). The increasing availability of fluconazole as well as the advent of highly active antiretroviral therapy for human immunodeficiency virus (HIV)-infected patients have decreased the incidence of cryptococcal infections.

While immunosuppression due to HIV is most classically associated with cases of cryptococcal infection, medication-induced immunosuppression is an increasingly recognized etiology of various types of cryptococcal infections (12). The immunomodulatory agent used to treat relapsed-remitting multiple sclerosis (MS), fingolimod, has been associated with at least 12 case reports to date of different types of cryptococcal infections, including primary meningoencephalitis (13–17) and primary cutaneous cryptococcosis (18–21).

We report a case of biopsy and culture-proven cryptococcal chest wall mass with pathologic fracture of rib in a patient on long-term fingolimod therapy for relapsed/remitting MS. Our case report represents a unique presentation of cryptococcal infection and highlights the importance of medication-induced cryptococcal infections and their subsequent management.

## Case presentation

**Figure 1:**
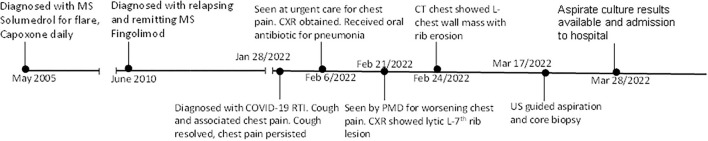
Timeline of progression of illness and workup.

**Figure 2:**
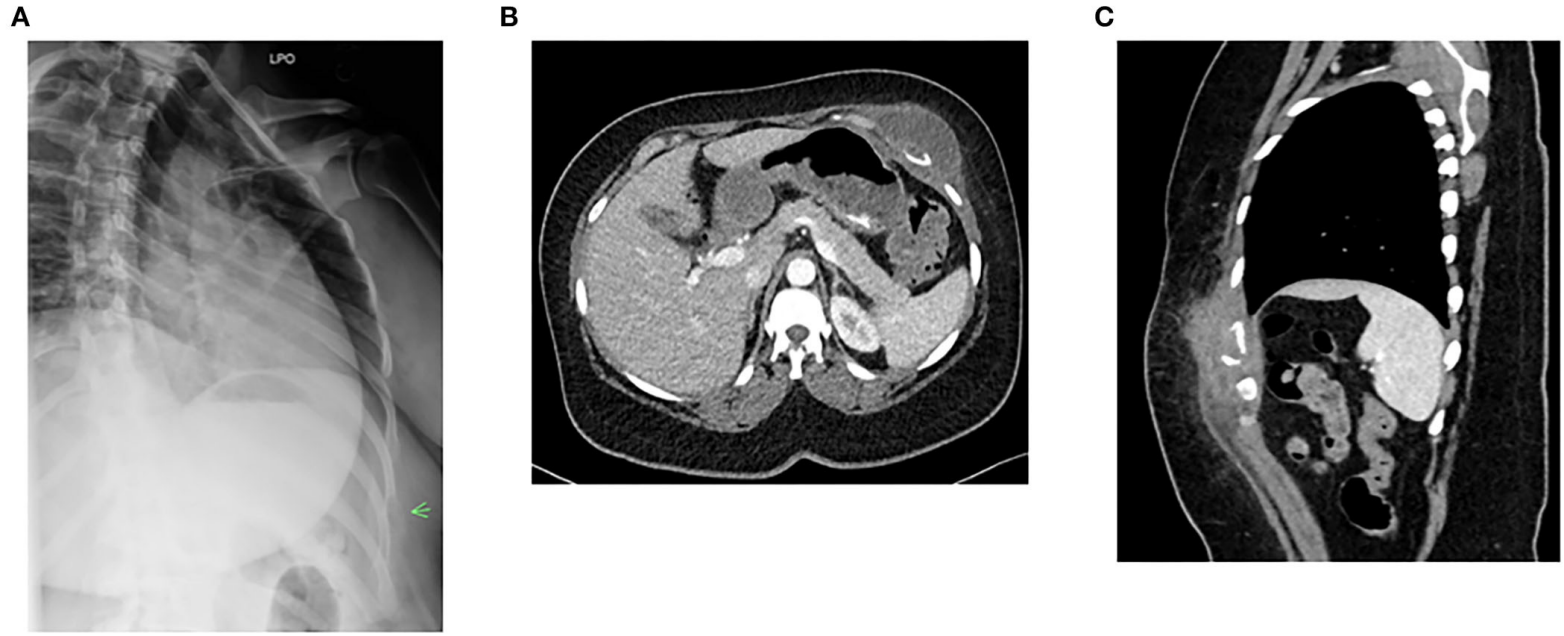
Imaging findings of chest wall mass and rib osteomyelitis. **(A)** Chest X-ray shows destruction of a rib. **(B)** Left chest wall mass (axial image). **(C)** Left chest wall mass (coronal view). Green arrow indicates the level of 7th rib fracture and mass.

**Figure 3:**
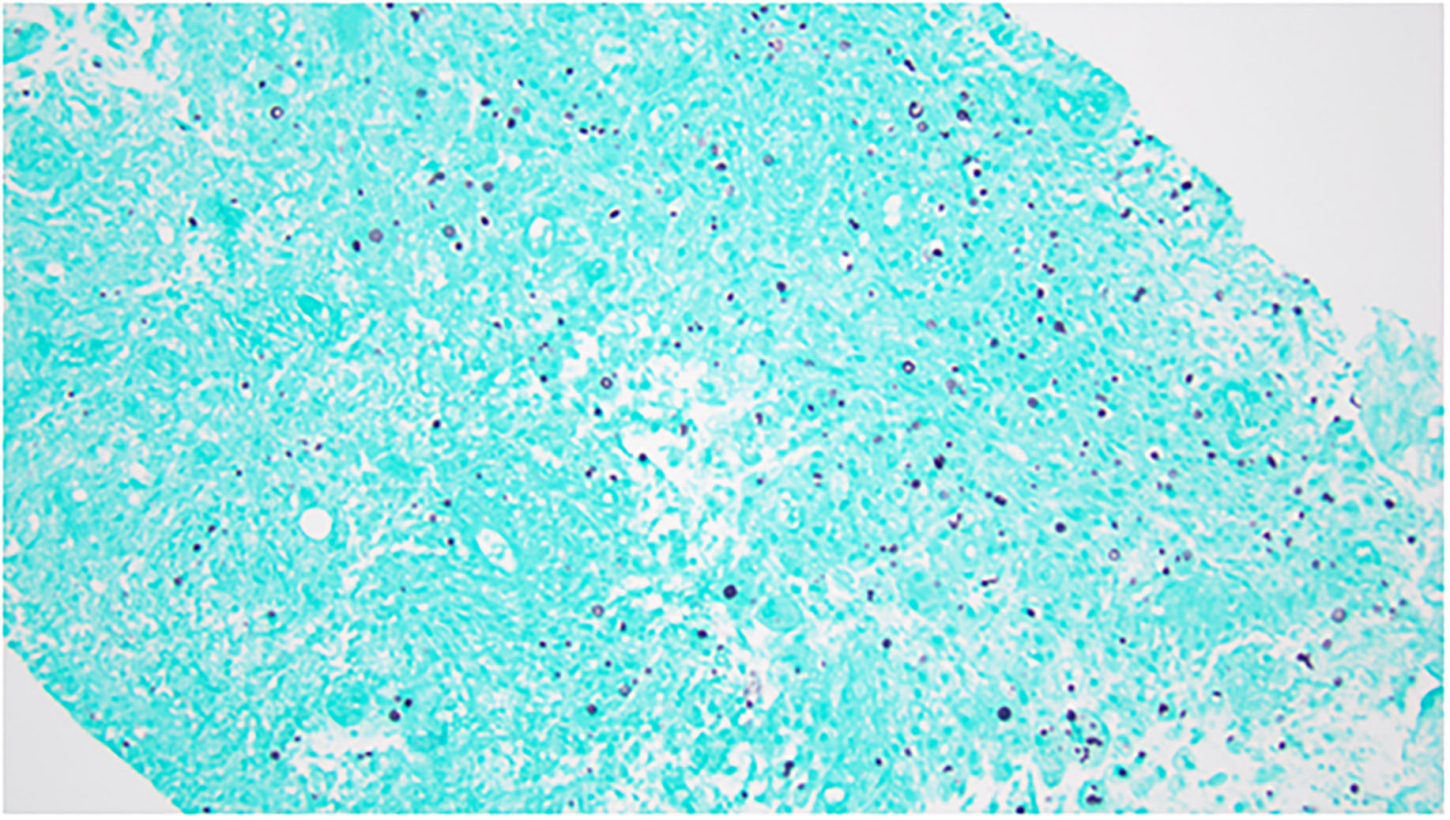
Grocott's Methenamine Silver (GMS) stain of aspirate showing yeast forms. Objective: 200×.

A 46-year-old female patient with medical history of relapsing-remitting MS who has been on fingolimod for more than 12 years was admitted on March 28 for left-side chest pain since the end of January. The pain was a dull aching type, becoming worse with deep breathing and touching. The timeline of her illness and workup is summarized in Figure 1. MS was diagnosed in May 2005 after presenting with numbness below the waist, loss of sensation in both of her feet, recurrent falls, and balance issues. At that time, she was treated with intravenous methylprednisolone for the initial flare, and a glatiramer daily injection was started after the initial flare. Her symptoms subsequently resolved on this therapy. She did not have any additional flares until the end of 2009 when she presented with problems of walking and lower extremity weakness. She was hospitalized and treated with methylprednisolone for an acute MS flare. At that point, she was considered to have relapsing, remitting MS. When she followed with neurology again in early 2010, her MS therapy was switched from glatiramer to fingolimod. Since that time, she has not had another MS flare and continued to tolerate fingolimod well. In January 2022, she developed cough and chest pain and was diagnosed to have COVID-19. On February 6, she was seen at urgent care for left-side chest pain. A chest X-ray was obtained and she was told that she had pneumonia. She received a single dose of dexamethasone and an oral antibiotic. Her cough resolved in about 2 weeks. However, 3 weeks after the onset of illness, she was seen by her primary care physician for worsening left lower anterior chest pain. A chest X-ray obtained at that time showed a lytic lesion of the left 7th rib with no acute pulmonary process (Figure 2A). On February 24, computerized tomography (CT) of chest was obtained and showed left chest wall mass with an erosion of left 7th rib subtle pathologic fracture. On March 17, ultrasound-guided aspiration pus and core biopsy of left chest wall mass were obtained. Gram stain and AFB smears were negative but Grocott's Methenamine Silver (GMS) stain showed “yeast forms” (Figure 3). Aerobic bacterial cultures and fungal cultures grew *C. neoformans*. On March 28, culture results were released and the patient was admitted to hospital for further management.

On admission, her home medication list did not include disease-modifying agents other than fingolimod. The patient also denied receiving steroids in the past at least 10 years other than a single dexamethasone injection she received 7 weeks prior to hospital admission. Her physical examination showed normal vital signs and a violaceous left anterior chest wall mass (approximately 5.5 cm length× 1.5 cm width) above the left 7th rib. The mass was soft and tender to palpation. CT of chest and abdomen showed left chest wall mass and destruction of 7th rib (Figures 2B,C). The patient was diagnosed to have cryptococcal chest wall mass with rib osteomyelitis and pathologic fracture. Further workup showed that HIV antigen and antibody test was negative and serum cryptococcal antigen titer was 1:80. Complete blood counts showed a total lymphocyte count of 300/μl (normal range: 1,100–3,900/μl) and complete metabolic panel was normal. Magnetic resonance imaging (MRI) of the brain showed previously known MS changes. This was followed by a lumbar puncture. The opening pressure was normal. Cerebrospinal fluid (CSF) cell counts, protein, and glucose were normal. CSF cryptococcal antigen and fungal culture were negative. As chest wall mass from *C. neformans* is very rare, the isolate was genotyped using the methods described previously and compared with other isolates (22). Supplementary Figure 1 performed shows that the isolate from our patient was serogroup A.

The patient was evaluated by a thoracic surgeon and interventional radiologist for possible debridement or placement of a drainage tube. There was no drainable residual abscess and extensive surgery was deemed unnecessary at this time. On admission to the hospital, fingolimod was discontinued. Due to the lack of literature on the management of extensive soft tissue and bone cryptococcal infection associated with fingolimod, we decided to start with the initial intensive treatment. The patient received 2 weeks of intravenous liposomal amphotericin B and flucytosine, followed by oral fluconazole with a plan to continue for 6–12 months. The patient has been adherent to medication. At 6 weeks of follow-up, chest pain has resolved and a follow-up CT showed a marked decrease in the size of chest wall mass.

## Discussion

Disease-modifying agents, including fingolimod, are used frequently and have been shown to reduce long-term disability in patients with relapsing-remitting MS. However, these agents are associated with a significant risk for opportunistic infections, including cryptococcosis with an incidence rate of about 9 per 100,000 person years (23). To date, we have noted 18 case reports of cryptococcal infections associated with the use of fingolimod, recently reviewed by Ma et al. (15) and at least four other new reports (19, 21, 24–26). These cases have described primary CNS cryptococcosis, primary cutaneous cryptococcosis, pulmonary cryptococcosis, and disseminated cryptococcosis (19, 21, 24–27). Among the 18 cases reported, 6 had subacute to chronic skin and/or soft tissue lesions (Table 1). The skin and soft tissue cryptococcal lesions reported in association with fingolimod include erythematous nodule from disseminated cryptococcus (29), ulcerative lesion from disseminated cryptococcosis (30), primary tender nodule (20), and primary non-healing ulcerated lesions (19, 21, 28). Our case is the first report of a cryptococcal chest wall mass with rib osteomyelitis following the use of fingolimod. Inflammatory pseudotumor responses have been reported in HIV-infected patients with disseminated cryptococcosis (31). Our case is HIV-negative and does not have the evidence of dissemination. The presence of a small cavitary pulmonary nodule in the left lower lobe raises the concern of direct extension to the chest wall, forming empyema necessitans.

**Table 1:** Clinical characteristics, treatment, and outcome of patients with cryptococcal skin and/or soft tissue infection associated with the use of fingolimod.

**References**	**Clinical findings**	**Absolute lymphocyte count (per uL)**	**Duration of fingliomod use**	**Work up to establish skin and soft tissue cryptococcosis**	**Treatment**	**Outcome of skin lesion**
(28)	Left shoulder ulcerative lesion of several months duration	300 (CD4 73, CD8 19)	2 years and 5 months	Histology and serum cryptococcal antigen	Fluconazole 400 mg daily for 6 months, 200mg daily for 6 months	Skin lesion healed within 2 months of treatment
(20)	Tender nodule on the forehead of 3 weeks duration	650 (CD4 56, CD8 121)	3 years	Biopsy culture grew C. neoformans	Fluconazole 800 mg loading dose, followed by a plan to continue 400 mg daily for a minimum of 6 weeks	Lesion healed with a scare at 1 month of treatment
(19)	Skin ulcer of upper thigh of 2 years duration	300	9 years	Positive PCR on biopsy	Fluconazole 600 mg twice daily for 14 days followed by 400 mg twice a day for 4 months	Lesion healed
(21)	Occipital ulcerated plaques of 4 years duration	Total not available (CD4 13, CD8 147)	7 years	Biopsy culture grew C. neoformans	Fluconazole 400 mg daily for 6 months	Healing at 3 months
(29)	Erythematous nodule (with subsequent ulceration) under the lower lip of 3 months duration^a, b^ [other organs involved: Lung and CNS]	300 (CD4 145, CD8 113)	2 years	Histology, skin biopsy culture grew C. neoformans CSF Cryptococcal antigen of 1:1024	Liposomal amphotericin B and flucytosine for 6 weeks followed by 8 weeks of fluconazole 400 mg daily and maintenance therapy	Skin lesion almost completely healed after 6 weeks of induction treatment
(30)	Headache of 2 weeks duration, facial ulcerative skin lesion^b^ [CNS]	500	3 years and 5 months	MRI showing meningeal enhancement and mass lesions, skin histology, CSF cultures grew C. neoformans, Cryptococcal antigen of 1:108 and 1:128 in CSF and serum, respectively	Liposomal amphotericin B and flucytosine for a total of 8 weeks followed by fluconazole	Improved and stable at 4 months of treatment
Our patient	Chest pain and mass of 2 months duration	300	>12 years	Aspirate culture grew C. neoformans, core biopsy histology showed yeast with acute inflammation, serum cryptococcal antigen of 1:80	Liposomal amphotericin B and flucytosine for 2 weeks followed by fluconazole 400 mg daily	Chest wall mass resolved and lymphocyte count normalized.

All patients were HIV-negative. Skin biopsy was obtained to establish diagnosis and fingolimod was topped once cryptococcosis was diagnosed in all patients.

^a^Patient had findings suggestive of pulmonary involvement.

^b^Patient had findings suggestive of CNS involvement.

The exact mechanism of fingolimod's effects on the risk of acquisition of cryptococcal infection remains unclear. One review suggested *C. neoformans*' unique ability to establish latent infection and evade immune escape mechanisms, combined with fingolimod's suppressive effects on multiple lines of the immune system needs further investigation (32). Another study in the murine model hypothesized that the reactivation of cryptococcal granulomas following the administration of fingolimod could be due to multiple mechanisms including profound CD4 and CD8 T-cells depletion, decreased macrophage phagocytosis, and decreased production of reactive oxygen species by macrophages (33).

A phase 3 clinical trial on fingolimod and a study after its introduction for clinical use showed that fingolimod causes lymphopenia in 8–13% of patients (34, 35), mostly within the 1st year of use (36). This is not surprising because fingolimod, a sphingosine-1-phosphate receptor (S1PR) modulator, works by reducing the recirculation of C-C chemokine receptor type 7+ (CCR7^+^) lymphocytes (37) and this may have relevance for protection against cryptococcal infection. Fingolimod does not affect CCR7+ T cells that include peripheral effector memory T cells and had no marked effect on T cells' ability to produce IFN-γ (37) and, therefore, the lymphopenia may not be severe enough to increase the risk of latent tuberculosis infection (LTBI) or affect the results of IFN-γ-based tests for LTBI (38). The effect of fingolimod on lymphocyte counts appears to be reversible but may take several months after holding the drug (39). As red blood cells (RBC) are the main regulators of serum sphingosine-1 phosphate concentration, a decrease in the number of RBCs may affect the severity of fingolimod-associated lymphopenia and reversal of counts (40).

It is believed that cryptococcal isolates vary in their virulence and tissue predilection. For instance, capsule-deficient Cryptococci cause a focal or dispersed granulomatous inflammatory reaction with areas of necrosis and minimal suppuration (41). The *C. neoformans* isolated from our patient was mucicarmine positive and, therefore, unlikely to be capsule deficient. Furthermore, genotyping study showed that the cryptococcal isolate was serogroup A (Supplementary Figure 1).

Due to the rarity of the cases of isolated cryptococcal infection associated with fingolimod use, no definitive treatment guidelines or consensus exist aside from those proposed in various case reports. We elected for a more aggressive treatment approach due to pulmonary and skeletal involvement and patient's immunocompromised state.

Due to increasing reports of fingolimod-associated cryptococcal infections, we believe it is important for MS and other neurological care providers to be aware of the risks associated with such immunomodulatory therapies. The features of our case and previously reported cryptococcosis highlight the need for future studies on the immunosuppressive effects of fingolimod and the exact mechanisms of how it increases the risk for opportunistic infections including cryptococcosis. Our case further highlights the need for a multidisciplinary team including internists or infectious disease specialists in conjunction with neurologists, thoracic surgeons, and interventional radiologists as the best approach to managing complex cases such as we have presented.

The strengths of this article include (i) description of a unique case of soft tissue and bone cryptococcal infection in a patient who was on fingolimod, (ii) presentation of follow-up data on clinical and radiological responses, while a patient is on anti-fungal treatment, and (iii) review of the literature with a focus on soft tissue cryptococcal infection and fingolimod. The limitation of this article includes difficulty in establishing a definite causal association between fingolimod and cryptococcal infection. However, the timeline and absence of other risk factors suggest that fingolimod is the likely risk for cryptococcal infection in our patient.

## Conclusion

We reported an atypical and aggressive form of cryptococcal infection in a patient who presented with left-side chest pain and left chest wall mass possibly related to immunosuppression from fingolimod use. Discontinuing fingolimod and antifungal treatment led to clinical improvement and marked a decrease in the size of chest wall mass.

## Patient perspective

The patient is happy with the care she is receiving. Her chest pain resolved but developed generalized itching after the initiation of fluconazole. On 6 weeks of follow-up visit, she agreed with the change of treatment to itraconazole.

## Data availability statement

The original contributions presented in the study are included in the article/Supplementary material, further inquiries can be directed to the corresponding author/s.

## Ethics statement

The patient provided written informed consent for performing genotyping studies on cryptococcal isolate and publishing all relevant clinical results.

## Author contributions

KC, MK, VC, and KT prepared a draft manuscript. GA, AE-D, RC, MD, BM-W, and ER revised the manuscript. MD and BM-W performed genotyping of cryptococcal isolate.

## Conflict of interest

The authors declare that the research was conducted in the absence of any commercial or financial relationships that could be construed as a potential conflict of interest.

## Publisher's note

All claims expressed in this article are solely those of the authors and do not necessarily represent those of their affiliated organizations, or those of the publisher, the editors and the reviewers. Any product that may be evaluated in this article, or claim that may be made by its manufacturer, is not guaranteed or endorsed by the publisher.
