# Supplementary figures and images for: Cryptococcal chest wall mass and rib osteomyelitis associated with the use of fingolimod: A case report and literature review
Source: Front Med (Lausanne). 2022 Sep 7;9:942751. doi: 10.3389/fmed.2022.942751 (PMC9491343; doi:10.3389/fmed.2022.942751)

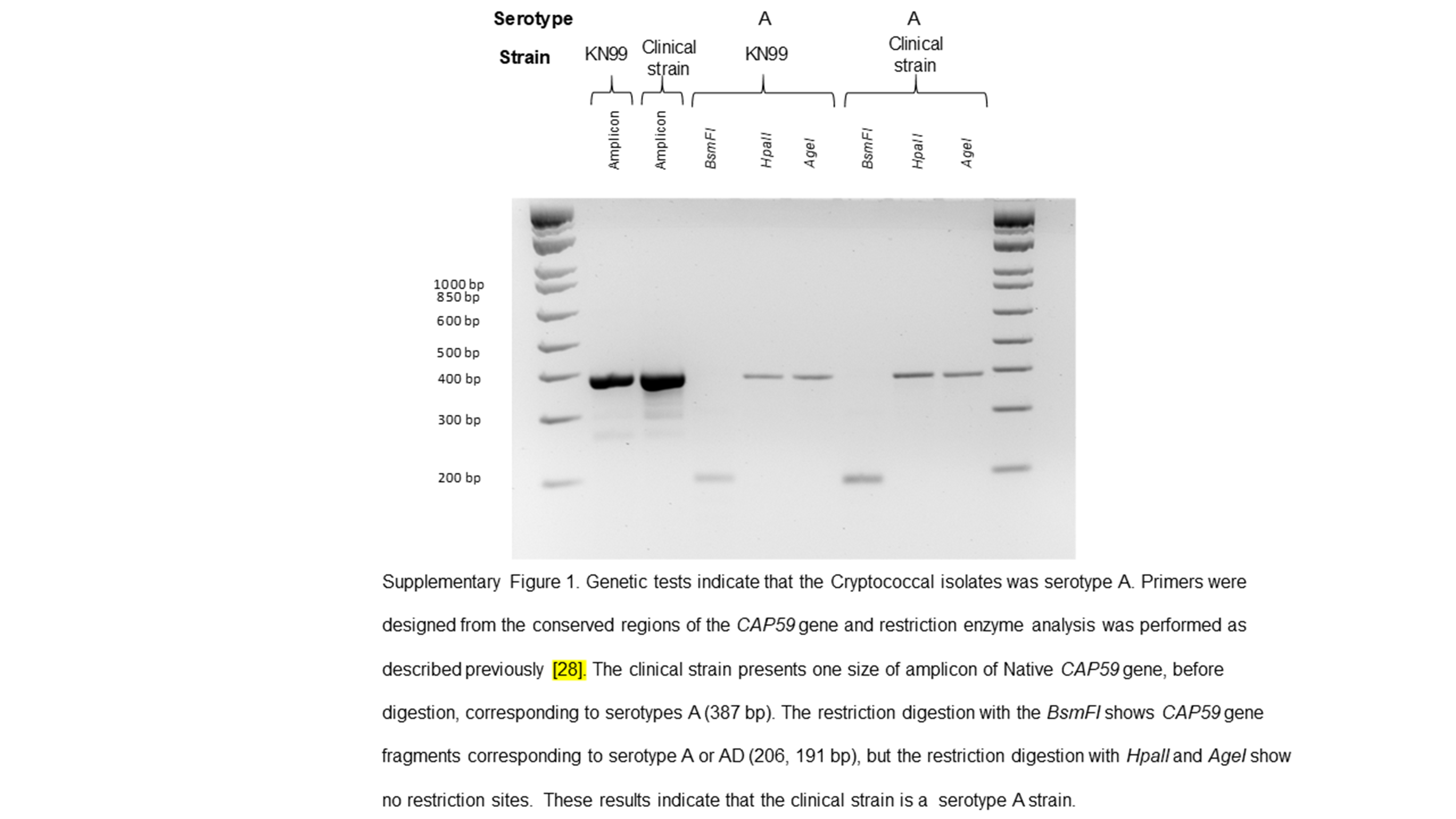

Supplement: Supplementary file 1 [file Image_1.TIF]
